# Phylacogen in the Treatment of Diseases of the Eye

**Published:** 1915-08

**Authors:** V. A. Decot

**Affiliations:** Buffalo, N. Y.


### Phylacogen in the Treatment of Diseases of the Eye.

By V. A. DECOT, M. D., Buffalo, N. Y.

Many diseases of the eye, because of the constitutional factor back of them, are most difficult to treat, especially those dependent upon gonorrheal and rheumatic infection. It is impossible to benefit the ocular disorder until the constitutional disease, whatever it may be, has been successfully controlled. The classical internal treatment with sodium salicylate and like drugs has been of considerable value especially in eye diseases based on true rheumatic infection, but for cases dependent upon gonorrhea and ordinary non-specific infections the lack of a specific remedy has been keenly felt. For that reason any preparation giving promise of dependable action against the cause of an iritis or conjunctivitis or other disease of the eye will be most heartily welcomed; and I believe, from my experience in over a hundred cases, that we have such a product in Phylacogens.

Of the many cases of gonorrheal iritis and conjunctivitis, rheumatic iritis, post-operative iritis following cataract extraction, ophthalmia neonatorum, and similar diseases which I have treated with Phylacogen in the past few years, the great majority have been entirely successful, and the treatment has come fully up to expectations. The percentage of cases in which relief was prompt and certain was much greater than that obtained after the usual remedies, and benefit was obtained under conditions that left little doubt that the results might properly be credited to Phylacogen therapy.

The Phylacogens have been at the command of the medical profession a sufficient length of time so that most, if not all, physicians are more or less acquainted with their composition, their indications and the theory upon which their administration is based. They are not a sterile suspension of bacteria in physiologic salt solution like the bacterial vaccines; but are formed by the growth of micro-organisms on proper culture media. The germ bodies are discarded, and the filtrate, properly prepared, is what is made use of in the treatment of disease under the name Phylacogen. Mixed Infection Phylacogen is a polyvalent product of several bacteria met with in the common mixed infections; while Rheumatism Phylacogen and Gonorrhea Phylacogen differ from this basic preparation in that they contain fifty per cent. of the metabolic products of the Streptococcus rheumaticus of Poynton and Paine and of the Gonococcus respectively.

The Phylacogens are administered in larger doses than are the bacterial vaccines, and are at times given intravenously to advantage, a procedure never resorted to in vaccine therapy.

Five to ten cubic centimeters of Phylacogen, injected subcutaneously, is not an excessive dose, while the intravenous dosage varies from a fraction of one cubic centimeter to as high as five. One or two c. c. may be administered subcutaneously as a primary dose in an adult and increased from day to day; although I have given this same dose to an infant, five days old, without the slightest ill effects, as will be made clear, in one of the cases that I wish to report.

Following a subcutaneous injection of Phylacogen, the site becomes reddened and tender in a few hours. This is not due to infection, but is rather to be viewed as a specific reaction of the surrounding cells in performing their function of preparing protective substances for use of the blood to destroy whatever bacteria may be present in the body from the original disease. In a rare case the patient complains of this local reaction as exceedingly annoying, But in the majority of instances the part into which injection is given is favored for a day or two and little complaint is made.

After an intravenous dose, or a large dose subcutaneously, what is known as a general or constitutional reaction ensues. This is manifested by chilliness or a distinct chill, rise in temperature and in pulse-rate, nausea and occasionally vomiting, shooting pains, etc. While many cases are cured without evidence of this reaction at any time, yet there are others which show a rapid stride in improvement immediately after a pronounced chill and increase in temperature. And this sudden improvement sometimes takes place after previous, reaction—free Phylacogen treatment has apparently accomplished little. I have never seen untoward results from these reactions, and simply ignore them. The patient, too, as a rule, pays little attention to them, especially when decided improvement is manifested.

It is not intended that Phylacogen treatment shall entirely displace local treatment. The installation of atrophine solution in iritis, for example,, is very necessary for its mechanical effect on the Iris. The same is true of hot and cold applications, which alter the blood supply of the diseased parts and remove the possibility of stasis, at the same time bringing to the field fresh rich blood probably loaded with anti-bodies created by the administration of Phylacogen. The employment of argyrol solution is also advisable when there is superficial inflammation, as this doubtless destroys the infection that is so superficial as to be beyond reach of the blood but nevertheless capable of constantly reinfecting the deeper tissues. Other classical measures in the treatment of ocular diseases should be employed as indicated. In this way the phylacogens are allowed to exert their full effect and I have found that excellent results are as a rule prompt in appearing, provided the etiological factor is discovered and the proper Phylacogen administered.

A striking feature of many of my iritis cases in which synechiae had formed has been the case with which atropine dilates the iris after one or more doses of Phylacogen, when previously the drug had little effect. This fact seems to indicate that the adhesions are made less tenacious when the localized infection has been vigorously attacked by the antibodies in the blood.

The disappearance of the dense capsule after cataract extraction, rendering a needleing unnecessary, in cases treated with Phylacogen, is likewise rather surprising but quite constant. An example of this is mentioned in the accompanying case-reports, and at the same time indicates the value of this treatment in post-operative iritis, which is an exceedingly serious complication so far as the integrity and function of the eye is concerned.

The following reports indicate what may be expected from Phylacogen therapy in different diseases of the eye, and give in more or less detail treatment that I have found exceptionally successful in a rather extensive series of cases.

Case 1. Mrs. M. Age 40.

Diagnosis: Double Iritis.

Mr. M. came to me after two weeks treatment at the hands of his lodge physician. Both irides were bound down, with extreme redness and pain. Atropine had not been used as he had been treated for ''pink eys.'' I used atropine; the pupils did their best to dilate and the pain was almost unbearable. I used leeches and weakened the atropine solution as the pain was so severe after its use. On the fourth day I persuaded the patient to allow me to use Phylacogen. I could get no history of either true rheumatism or gonorrhea, but directed his physician to give Rheumatism Phylacogen in 5 Cc. doses every other day. The pain disappeared after the third dose and the eyes improved steadily from that time. After five more 5 Cc. doses, the case cleared up remarkably well. Both irides were in fair condition; there was a little adhesion in both but not enough to impair the sight. I was unable to make a definite diagnosis, but the case cleared up promptly under the Rheumatism Phylacogen. The patient is a very fleshy person, full blooded, and appears to have a tendency toward rheumatism or gout.

I advised a continuance of his treatment to avoid relapse, but the patient refused on account of the local reaction. He made more complaint about the reaction at the site of injection than any other patient I have ever had. He appeared to have quite severe local reactions, but they cleared up after forty-eight hours. He had no systemic reactions, except following the last dose, at which time he had chills and fever. His physician reported that he had difficulty in keeping his patient from the overuse of alcoholic stimulants at times.

Treatment:—5 Cc. Rheumatism Phylacogen and 1 Cc. Adrenalin Solution (1-3200) were given for three doses, I then gave Rheumatism Phylacogen 10 Cc., for three more doses. Patient had marked reaction after each 10 Cc. dose, severe chills and fever.

Results: Case cleared up entirely, no adhesions of iris, all old adhesions became loosened.

Case 2. Mr. J. E. L. Aged 60.

Diagnosis: Iritis following operation for cataract.

History: Patient had a cataract, had lost one eye by operation some five years ago, probably due to iritis setting-in after a successful cataract operation by some other physician.

I removed the cataract in the remaining eye and sent the patient home at the end of the second week. A few days later he returned with beginning iritis. All physicians who have performed cataract operations know how serious this condition is and how poor the chances are for recovery; those who have never experienced this trouble have not done their share of cataract operations.

Treatment: I at once used the Rheumatism Phylacogen, first giving 5 Cc. every day, then 10 Cc. with Adrenalin every 3rd day; and not only did the iritis subside but the capsule which seemed so thick became transparent. I have since fitted the patient with glasses without doing a needleing. Patient had a severe reaction after each 10 Cc. dose.

Result: Patient has 2/3 normal vision and a good sized pupil.

Case 3. Mrs. M.

Diagnosis: Iritis, following operation for cataract.

History: Mrs. M. has only one eye; that eye had cataract. I operated. Three weeks after operation she developed iritis, a serious condition, especially with a dense capsule. This is my second case with a similar history, and I decided to try Rheumatism Phylacogen in her case.

Treatment: I gave 5 Cc. every other day for the first week. I then used it in 10 Cc. doses. The reaction was very severe following the second 10 Cc. dose, so severe in fact that the patient would not allow me to use any more. The pain had ceased, however, and I am glad to say the pupil, while not what I wished it to be, is large enough to allow for refraction. And, strangest of all, the capsule thinned out so that a needleing was not necessary. This may of course have been secretion or cortex, but we all know how we dread to see that tough capsule form, and anything that in any way lessens the difficulty is surely welcome. As I have said this is only my second case of this kind, but I am going to watch the effect in my future cases. If this result should prove constant, it might be advisable to give Phylacogen before operation in suspected cases of rheumatism and in cases that give a gonorrheal history. It has surely stopped the advance of the iritis in these two cases and that was what was desired.

Patient had been treated for months for syphilitic iritis, without any lasting benefit.

I confirmed the diagnosis, but was not satisfied with the history of the case and the reported treatment, mercury, etc.; hence, I prescribed mercury hypodermically. The result from this was apparently good; that is the pain lessened, but the iris adhesions would not give way, and she came back three times with a recurrence of all symptoms. I had her physician try the Rheumatism Phylacogen, and for a time it seemed to help her, but her symptoms returned. I then advised a liberal dose of the Mixed Infection Phylacogen and was surprised to see the remarkable effect in a week. Her physician reported that he used 5 Cc. Mixed Infection Phylacogen every day, with little reaction. The case has cleared up entirely; the adhesions have not entirely disappeared but are much improved and so far there has been no signs of a relapse. This case was treated in Sept. 1912.

I have had similar results since, that is in cases in which I was almost sure that the primary cause of the trouble was syphilis; but the inflammation of the iris yielded readily when I used Mixed Infection Phylacogen. Another interesting feature is that in cases that seem a little slow in getting results from the Phylacogens, very marked improvement follows immediately when a reaction occurs, (chill, nervous shaking, fever and sometimes shooting pains).

Case 5. Mr. A. E., aged 38, clerk in railroad office.

Diagnosis: Rheumatic iritis.

History: Patient gave history of semi-annual attacks of apparent iritis; no certain diagnosis could be made in the past; and I found it exceedingly hard to give a definite diagnosis. The attacks began as a slight conjunctivitis, which later developed into a very severe attack of iritis; the iris became adhesive and the entire surroundings gave the appearance of a deep cellulitis.

Treatment: I instructed his physician to give 5 Cc. Rheumatism Phylacogen daily for three doses, and then 10 Cc. every three or four days. The patient showed improvement but threatened relapse when we tried to discontinue the Phylacogen. He had very severe reactions after each dose, was obliged to go to bed several times; but after fifteen doses he was entirely cured. He was back to work in a week, but the eye was not really cured until after his fifteenth dose. Atrophine was used to keep the pupil open.

Results: Patient is entirely cured, and has passed two of his "iritis seasons" as he calls them.

Case 6. Master B., aged 11.

Case of corneal ulcers, following an attack of measles. Patient also had an infected ear, an infected nose, and a sore on his finger that would not heal. He would not eat, was restless at night, and had swollen glands in the neck. I used atropine in both eyes, advised hot applications, and gave 3 Cc. Mixed Infection Phylacogen. Improvement was very noticeable after the third injection. I then gave a 5 Cc. dose but the boy developed a temperature of 101, and I suspended the Phylacogen until the temperature became normal. I then gave it in 2 Cc. doses and the child steadily improved; the ulcers of the cornea cleared, the nose and the finger became entirely well. The enlarged glands, while not entirely normal in size, are hardly larger than a pea. His mother says the boy eats, sleeps, and acts better in every way. I have three other cases similar to this one, being treated by the family physician, with the same treatment. It has done so well in these cases that I wonder if it would not be beneficial in the treatment of measles. I believe it would be worthy of trial.

Case 7. Mr. Ed. B., aged 50.

Mr. B.'s case was treated at the dispensary for two weeks before coming under my care. He suffered all the symptoms of iritis, very severe pain, etc. One eye was turned inward and upward and had been defective since boyhood. It appeared to be a case of sympathetic trouble but there was no history of accident. Atropine and dionin had been employed. I used leeches, gave sodium salicylate, etc. After the second day I gave 5 Cc. Rheumatism Phylacogen and repeated it on the third day. I did not get the usual good results; so on the fifth day decided that I would give Mixed Infection Phylacogen, as the patient remembered that his trouble began shortly after caring for a horse that had skin trouble. I reasoned that the condition may have been caused by an infection of the conjunctiva, which later involved the ciliary region, as the eyes were very tender when pressure was exerted over this area. I noticed very marked improvement before the third dose of 5 Cc. Mixed Infection Phylacogen, and advised ice applications, argyrol three times a day and atropine. The patient had marked reactions in the form of chills after each injection of Phylacogen. After the fourth dose he felt so much better that he would not permit the use of any more Phylacogen. The case then progressed more slowly, but both eyes finally cleared up perfectly. I believe that this iritis was caused by an infection, and had progressed to an irido-cyclitis. The Mixed Infection

Phylacogen should certainly be credited with its speedy cure.

Case 8. Mrs. V. B., age 40.

I was called by Dr. N. to see a case of double gonorrheal iritis with purulent conjunctivitis, the patient also had a gonorrheal urethritis. I ordered her sent to the hospital, ice bags applied to the eyes, atropine instilled, with 50% argyrol solution alternating every hour night and day.

I also ordered 10 Cc. Gonorrhea Phylacogen given every other day regardless of the very severe reaction she showed after each dose. On the fourth day the swelling of the lids subsided to a very marked degree, the pus was much less, the iris fully dilated. The case steadily improved, and after the 15th dose of Phylacogen I let her go home.

There remained some slight conjunctivitis, with a trace of purulent secretion, for which I had her continue the strong argyrol solution. I also advised another one or two doses of Phylacogen. The patient was to report any signs of relapse. This case was treated in December 1912 and I have not seen or heard from her since. If there had been any sign of a return of the disease her physician would without doubt have notified me.

The case is quite remarkable, as gonorrheal pus in the eyes is usually fatal to the cornea, and not only did the eyes clear up but her primary trouble was completely cured.

Case 9. Chas. S. Baby—5 days old.

Diagnosis: Gonorrheal Ophthalmia Neonatorum.

History: The little patient's eyes were so puffed that when an attempt to open the lids was made the pus spurted out. The conjunctiva was swollen so that both eyes protruded. It was impossible to see the cornea. The pus was examined by microscope and found loaded with gonococci; later inquiries verified this as both parents were found to be infected, primary trouble being traced to the father. I cleaned the lids as well as possible and used as a cautery carbolic acid and nitrate of silver. I then gave 2 Cc. Gonorrhea Phylacogen subcutaneously. The reaction was very severe; the child had severe chills and a temperature of 104.

The third day I again burned the lids and gave 2 Cc. Gonorrhea Phylacogen subcutaneously; the reaction was slight. The infant was being treated at home with argyrol 50% every hour night and day and ice compresses.

On the fourth day I did not think it necessary to burn the lids, as I could see the cornea very easily, and the swelling had subsided. The secretion was very much less and not so thick.

At the end of the eighth day, the patient had improved so much that I stopped the use of ice and employed the argyrol only about four times a day. There was no corneal involvement, and the eyes are now normal in every respect. While I feel that the regular treatment was very essential, I also believe that the Phylacogen was a marked factor in the prompt and complete recovery; certainly in every other similar case I have had to burn the lids more than twice, and the burning I fear has often opened the way for corneal complications. The readiness with which this case responded to treatment, and the stopping of the formation of the pus must be attributed to the Phylacogens.

Case 10. Patient Mr. S. A., aged 30.

This was one of the worst cases of gonorrheal iritis I have ever seen. The patient had acute gonorrhea in a very virulent form; and gave a history of previous attacks many years before. He thought this was a relapse; but this, however, was the first time that his eyes had given him trouble. He was treated by a physician in his home town until both eyes were involved, the irides in both eyes were bound down, painful, and the patient could not bear the slightest light. The conjunctiva of both eyes was deeply injected. After three weeks of home treatment, I saw the case in consultation at the Sisters' Hospital, and ordered atropine, leeches, sodium salicylate, rest in bed in a dark room, etc. Five Cc. doses of the Gonorrhea Phylacogen were given every day until the patient began to get quite severe reactions. The first five doses did not seem to take hold of the case; I stopped the Phylacogen for three days, then gave 10 Cc. at a dose. After the third injection he had a rather severe reaction, but the eyes began to respond to the atropine, and the discharge from the primary lesion began to subside.

This patient was confined to his bed for two weeks, during which time I had given five 5 Cc. doses and three 10 Cc. doses. At the beginning of the third week he was able to open his eyes, could recognize people at the foot of the bed, and wanted to get up. I then gave 5 Cc. doses every third day, his condition improved rapidly, and, at the end of his fourth week, the man went home. I ordered the Phylacogen used about once a week until there would be no danger of relapse either in his eyes or in his primary infection.

Mr. A. has since been to see me and was fitted for glasses. Result is perfect sight, there are slight adhesions of the iris of one eye, but not sufficient to impair vision in the least. The other eye is perfect, with no adhesions at all. His primary gonorrhea has entirely disappeared and his general health is much improved.

A most interesting feature of the case was the disappearance of a cloudy film which seemed to run across from the edges of the iris, this secretion was very marked when I first saw the case.

